# Implementation of national recommendations for the care of Ukrainian refugee children in Switzerland: a survey of primary care pediatricians

**DOI:** 10.3389/fpubh.2025.1666753

**Published:** 2026-01-20

**Authors:** Fabienne Nicole Jaeger, Sarah Depallens, Marc Sidler, Noemie Wagner, Nicole Ritz

**Affiliations:** 1 Swiss Tropical and Public Health Institute (Swiss TPH), Basel, Switzerland; 2 Universitat Basel, Basel, Switzerland; 3 Migrationsambulatorium Erlenhof, Erlenhof Zentrum, Reinach, Switzerland; 4 pädiatrie schweiz, Swiss Society of Paediatrics, Fribourg, Switzerland; 5 Centre Hospitalier Universitaire Vaudois, Lausanne, Switzerland; 6 Kinderärzte Schweiz, Zürich, Switzerland; 7 Hopitaux Universitaires Geneve Hopital des Enfants, Geneva, Switzerland; 8 Paediatric Infectious Diseases Unit, Geneva University Hospitals and Faculty of Medicine, Geneva, Switzerland; 9 Universitats-Kinderspital beider Basel, Basel, Switzerland; 10 Children’s Hospital of Central Switzerland, Cantonal Hospital of Lucerne, Lucerne, Switzerland; 11 Mycobacterial and Migrant Health Research Group, University of Basel Children’s Hospital Basel, Basel, Switzerland

**Keywords:** Ukraine, pediatric, refugee, recommendations, healthcare, screening, implementation

## Abstract

**Objectives:**

This study aimed to evaluate the implementation of specific recommendations for providers caring for pediatric Ukrainian refugees new to Switzerland, including the care provided and the challenges faced.

**Methods:**

Pediatricians were invited via newsletters to participate in an online questionnaire.

**Results:**

A total of 111 primary care pediatricians (PCPs) were eligible for participation. Of these, 91% reported providing care for Ukrainian refugees. The vast majority (88.6%) were aware of the mentioned recommendations. The reported care included treating acute conditions (94.8%), ensuring vaccination coverage (89.7%), and performing new-arrival check-ups regardless of age (60.8%). The psychosocial situation of the child (67.0%) and the mental wellbeing of both the child and main caregiver (47.4%) were assessed, and preventive information (29.9%) was provided to a lesser extent. Tuberculosis risks were discussed or screened for by 73.2% of the participants, HIV by 58.3%, and Hepatitis C by 57.3%. The main challenges faced included lack of time (77.9%), language barriers (81.1%), organizing mental health care (65.5.%), different health(care) perceptions (65.6%), demanding attitudes (68.4%), and refusals of check-ups (30.8%) and vaccines (38.6%).

**Conclusion:**

Despite high levels of awareness of the recommendations, their implementation varied. Preventive care and mental health support require improvement. Care-enabling policies have been identified and are warranted.

## Introduction

The invasion of Ukraine by Russian forces on 24 February 2022 led to the displacement of more than 6 million people and a massive exodus of predominantly women and children to Western Europe. Within three years, by February 2025, more than 114,900 Ukrainian individuals had newly arrived in Switzerland seeking protection, with the majority arriving during the first months after the conflict began (1). This led to a considerable and sudden increase in the pediatric population in Switzerland, where primary care pediatricians (PCP) and, to a lesser extent, family doctors provide the first line of care. This includes check-ups, vaccinations, guidance, triage, and management of acute and chronic conditions that can be addressed at the primary care level. From a public health perspective, it is essential that new arrivals receive appropriate curative and preventive care, including care for acute and chronic conditions, prevention information, vaccinations, and screening for transmissible diseases, as pediatric migrants and refugees may face additional health risks and needs depending on the country of origin, transit experiences, personal risk factors, and circumstances of migration (2, 3). Mental health care needs, undetected visual or hearing impairments, higher rates of certain infectious diseases, anemia, vitamin D deficiency, insufficient vaccination coverage, and conditions addressable through health promotion—such as poor nutrition habits and cavities—are among the medical challenges common in child refugees (2, 4–8). For children and adolescents from Ukraine, mental health problems related to the circumstances of war and flight, as well as a higher prevalence of certain transmissible diseases—such as tuberculosis, HIV, hepatitis B, and especially hepatitis C—compared to Swiss children, were anticipated (9, 10). Furthermore, the Ukrainian vaccination schedule includes fewer vaccines, and the second dose of the measles vaccine is administered later, implying the need for additional vaccines for Ukrainian refugees to meet Swiss standards (9). Providing care for refugees may be challenging for healthcare providers and—especially due to the large numbers of new arrivals—for health services in general. To ensure optimal quality of care, taking into account the specific needs of Ukrainian minors newly arriving in Switzerland, recommendations were developed by the reference group on migrant health of the national pediatric society, pädiatrie schweiz, and by the working group of members of the pediatric infectious diseases group of Switzerland (9).

The recommendations were first published in English in an international peer-reviewed journal in May 2022 (9) to ensure international accessibility. A shorter version was published in the national medical outlet of the Swiss Medical Association to provide nationwide information to all registered physicians in the two most widely spoken languages, German and French (11). In addition, 1 page briefs in German, French, and English were published on the website of the national pediatric society^1^ and on the website of the professional association of German-speaking primary care pediatricians, Kinderärzte Schweiz (KIS, www.kinderärzteschweiz.ch). Both associations also informed their members about the recommendations via newsletters.

Despite the large patient numbers and the widespread publication of guidance, little is known about the awareness and implementation of these recommendations by PCPs, as well as the potential challenges faced in providing care for Ukrainian children and adolescents. This study aimed to (1) evaluate the implementation of the national recommendations, (2) assess care practices among PCPs, and (3) identify barriers to implementation. The findings may contribute to improved implementation of future recommendations and the provision of care for the next waves of refugees.

## Methods

An online survey among primary care pediatricians in Switzerland was conducted using a predominantly quantitative approach while allowing for a few free-text answers. The questionnaire was developed by members of the reference group for migrant health of pädiatrie schweiz, the Swiss national association of pediatricians, with inputs from Kinderärzte Schweiz (KIS). The survey was programmed on Findmind,^2^ a Swiss online survey tool, in German and additionally translated into French to increase outreach, as members of the Swiss national pediatric society can choose to receive information in either French or German. The questionnaire was piloted among German- and French-speaking PCPs. Links to the questionnaire were then sent, along with a brief introductory text, as part of the regular newsletters in September 2022 to all members of KIS and the national pediatric society. The exact number of members who received the questionnaire through the two newsletters is unknown. Of the more than 850 members of the German-speaking organization KIS, the majority are also members of the national society. At the time of the survey, the national society had 1,112 members identifying as primary care physicians, 721 of whom had opted to receive information in German. Descriptive analysis was performed using the integrated statistics tool of Findmind and Stata 16. Chi-squared tests were performed on the Likert scale values to assess significance regarding a random distribution of answers. We then additionally converted the Likert scale values to numeric values and assessed the mean and variance and presented the variables in decreasing order of the means, to demonstrate results according to relevance attributed by respondents. We also regrouped the response categories “relevant,” “rather often relevant,” and “very frequently relevant” into a single category, “overall relevant,” to present the percentages of pediatricians considering an item as relevant. The few free-text answers were thematically analyzed and summarized narratively. Ethics approval was obtained by the Ethikkommission Nordwest- und Zentralschweiz (BASEC-Req 2023-00582).

## Results

### Participants

Of the 137 respondents, 111 identified as PCPs and were therefore eligible for inclusion in the analysis. We excluded responses from retired pediatricians (2), school physicians (4), those reporting on behalf of a hospital (11), and individuals identifying as non-primary care physicians (9). The response rate for eligible physicians cannot be calculated conclusively as there are no figures available on double memberships. According to pädiatrie schweiz, the vast majority of KIS members are also members of the national society, making a response rate of approximately 10% to the questionnaire, published in the newsletter alongside other topics, likely. A total of 21.2% of the eligible participants worked in single practices, while the remainder worked in group primary care practices, which is similar to the distribution reported by the federal medical association (FMH) for pediatricians: 23% in single practices and the remainder in group practices (12).

### Care provided

Among the 111 eligible physicians, 102 (91.8%) reported providing care for newly arrived Ukrainian children and adolescents in their private practices. A total of three (2.9%) reported that they had so far limited their care to acute complaints, while 98 (96.0%) reported conducting screening, general assessments, and updating vaccinations.

**Figure 1:**
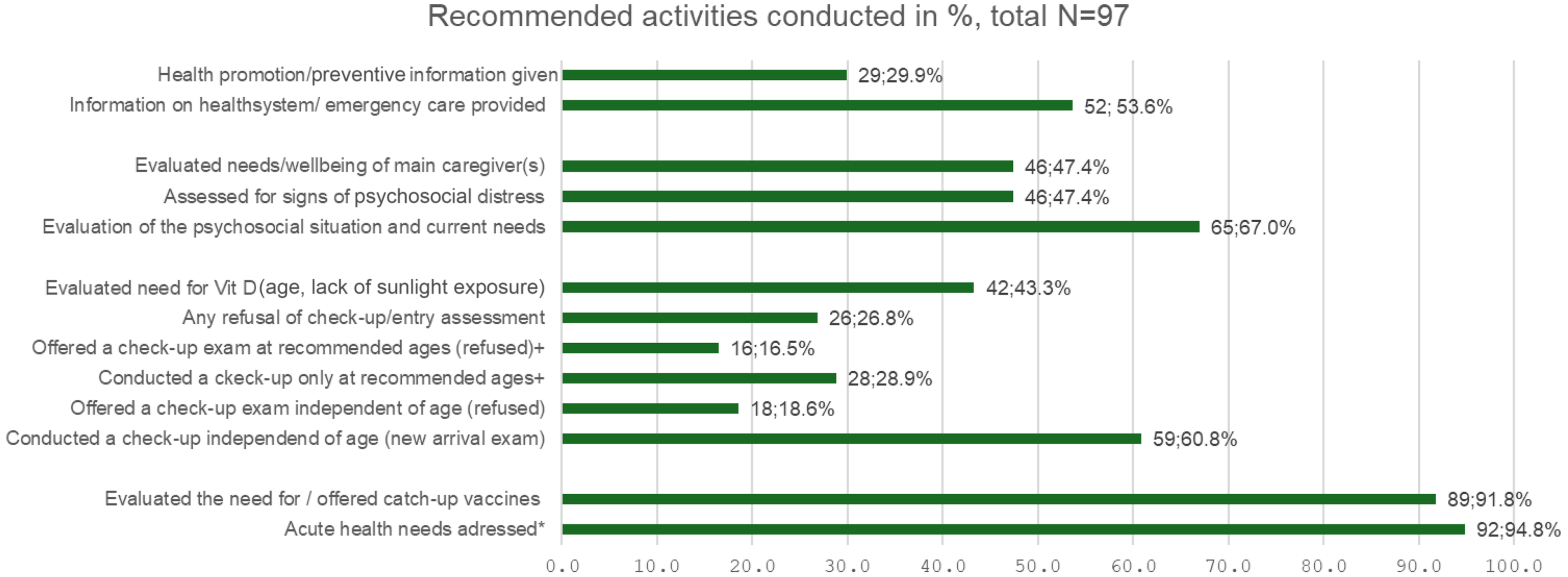
Recommended activities conducted. Number of primarycare pediatricians conducting recommended activity: total respondents 97 for this question, multiple answers possible. * acute complaints, medication for chronic medication prescribed etc., ^+^ according to national recommendations on the timing of check-ups for the general paediatric population.

Figure 1 provides an overview of the recommendations that have been most frequently followed for newly arrived Ukrainian children. The most frequently reported pediatric activities were as follows: (i) addressing acute complaints (e.g., acute sickness, medication for chronic conditions) (94.8%) and (ii) evaluating the need for catch-up vaccinations (89.7%). Early assessments are recommended by the guidelines, but our findings showed that only 60.8% of the physicians follow the recommendation to perform these assessments regardless of age. The remaining physicians at least conduct them according to the ages recommended for well-child visits by the national society for pediatrics (pädiatrie schweiz) for the general population. Implementation of this recommendation was further complicated by refusals encountered.

While psychosocial needs were assessed by 2/3 of the respondents, the mental health and wellbeing of the child and caregivers were clearly insufficiently addressed, with only half of the pediatricians reporting doing so. Furthermore, the recommendations to provide information on the healthcare system and age-appropriate preventive guidance were poorly implemented: 2/3 of the pediatricians reported providing some information on the healthcare system, and less than one-third provided preventive information.

Overall, only 11 of the 91 (12.1%) PCPs reported that vaccination documentation was always available. In the absence of such documentation, the majority reported (nearly) always trusting (15/80, 18.6%) or somewhat trusting (33/80, 41.3%) the vaccination history provided by the caregiver, whereas 28/80 (35.0%) reported that they rather did not trust it, and 2/80 (2.5%) reported never trusting the history given by caregivers.

The evaluated guidelines recommended offering general screening for tuberculosis, HIV, especially in the case of risk factors or the absence of negative maternal tests during pregnancy, and Hepatitis C. Table 1 shows the implementation strategies chosen by the participants: A substantial number neither discussed nor systematically screened for these diseases (see Table 1). Still, two-thirds of the participants discussed the risks of tuberculosis, and more than half discussed the risks of HIV and hepatitis C infection. Although systematic screening was generally low, it was highest for tuberculosis.

**Table 1:** Screenings offered in numbers and percentages.

Screening	*N*	Systematically done (*n*;%)	Always offered, individual risks discussed (*n*;%)	Offered selectively after discussion of individual risks (*n*;%)	No screening (*n*;%)
Tuberculosis	97	7; 7.2	15; 15.5	49; 50.5	26; 26.8
HIV-1/2	96	2; 2.1	14; 14.6	40; 41.8	40; 40.0
Hepatitis C	96	5; 5.2	13; 13.5	37; 38.5	41; 42.7

### Challenges faced

**Figure 2:**
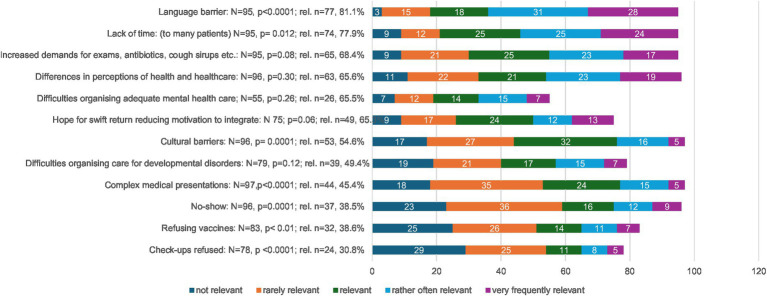
Challenges faced by primary care pediatricians. Rel = overall relevance, regrouping relevant, rather often, and frequently relevant. Significant differences were assessed as deviations from an expected random distribution.

The most relevant challenges reported while caring for Ukrainian pediatric refugees were the language barrier and lack of time, both of which were significant in the analysis (Figure 2).

Furthermore, the respondents also mentioned a demanding attitude with higher rates of expressed demands for exams (blood analysis, ultrasound etc.), and medications (antibiotics, antitussives etc.) compared to the autochthonous population and the differences in the perception of disease and health care.

### Implementation of recommendations

The PCPs were also asked whether they were aware of the existence of the recommendations. While the majority had read at least the summary of the recommendations (78/105, 74.3%) or knew of their existence (15/105, 14.3%), few (12/105, 11.4%) reported that they were not aware of their existence. Further challenges to the implementation of the national recommendations are summarized in Table 2 and include: (i) the knowledge and complexity of the recommendations, (ii) the need to draw blood and (iii) the parents refusing recommended testing.

**Table 2:** Recommendations: further barriers to their implementation.

Recommendations: further barriers to their implementation *N*=105	*N*	%
Not feeling familiar enough with the recommendations	15	14.2
Considering them too complex	13	12.4
Not feeling knowledgeable enough about catch-up vaccinations	13	12.4
Parents refusing blood to be taken	13	12.4
Difficulties in drawing blood	5	4.8
Children are healthy and do not need anything done	5	4.8
Children leave Switzerland soon anyway	1	1

In the free-text responses, further challenges were mentioned: Ukrainian individuals not being informed upon arrival in Switzerland of their right to consult a pediatrician of their choice; difficulties making appointments due to the language barrier; high levels of mistrust toward vaccines; no-shows and unpaid bills (refugees receive retrospective health insurance coverage starting from the day of registration in Switzerland, but administrative procedures may cause delays in payment); and parents perceived as demanding, requesting more examinations (e.g., blood tests, ultrasounds) and treatments such as antibiotics and antitussive medications more frequently.

## Discussion

This study provides an overview of the healthcare delivered to Ukrainian children and adolescents seeking protection in Switzerland. To the best of our knowledge, it is the first study specifically investigating pediatric primary care for Ukrainian refugees in a high-resource setting. It also highlights challenges in providing healthcare in the context of a sudden influx of new patients with a forced-migration background and different concepts of health and healthcare (13, 14). Furthermore, it evaluates the implementation of medical recommendations, indicating high awareness of their existence (>90%), most likely due to the use of multiple dissemination channels. The findings suggest that new information can successfully reach large numbers of physicians; however, implementation remains incomplete, especially regarding the assessment of mental health needs and the provision of preventive and healthcare information.

### Care provided

The study demonstrated that, in addition to addressing urgent medical issues, the pediatricians usually assessed the need for vaccinations, which is highly relevant from a public health perspective. It also showed that, although not always conducted as an entry examination as recommended, a substantial number of Ukrainian children and adolescents were offered check-ups. The challenges described, such as referring children to mental health or developmental specialists, demonstrated that patient needs were recognized but that addressing them adequately will require additional efforts from the health system. Discussion of risks and screening for infectious diseases that are more frequently encountered in Ukraine were conducted but not to the recommended extent.

### Challenges faced

The quality of care provided and the implementation of the recommendations must be assessed in light of the challenges associated with providing pediatric care for immigrants and refugees. A systematic review (15) and a multi-national European study (16) investigating the main challenges in providing healthcare to immigrants identified similar issues, including language barriers, continuity of care, differences in the perception of illness, and lack of healthcare coverage. While language barriers and differences in perceptions of illness were also relevant findings in our study, lack of healthcare coverage was not a relevant issue for Ukrainian minors in Switzerland, as mandatory health insurance for refugees ensures access to care. An older study focusing on PCPs providing care for migrants in various European countries also identified linguistic and cultural factors as the main challenges, with lack of access to interpreters—an issue that is also present in Switzerland (11)—and lack of guidelines and training in migrant health being highly relevant (17). Research specifically on the healthcare provided and the challenges met when caring for Ukrainian individuals has been mainly conducted in Poland, a neighboring country hosting large numbers of Ukrainian refugees (18–20), and the Czech Republic (21, 22), generally focusing on more than just the pediatric population. A Polish study including PCPs and adult healthcare providers, which focused specifically on challenges in caring for Ukrainian refugees, offered study participants only a limited set of response options in a multiple-choice format; difficulties related to the language barrier emerged as the most prominent finding (18). The lack of medical records and differences in medical products used in both countries may have been more relevant to providers caring for adults with chronic conditions (18). Missing access to medical records (16, 18) was not a questionnaire item in our study and was not mentioned in the free-text sections by our participants. In a qualitative study from the Czech Republic, the main challenges identified in the care of Ukrainian refugees were the language barrier, differences in healthcare systems, differing attitudes toward health and illness, and prejudices (21). Differences in the healthcare system and, consequently, the different position of the patient within the healthcare system were reported as the most important source of misunderstanding (21). Similarly, in our study, which focused only on pediatric primary care provided to Ukrainian refugees, language barriers and differing perceptions of health and healthcare were, next to lack of time, highly relevant challenges. Regarding vaccination hesitancy among Ukrainian refugees reported in the literature (10), nearly half of the respondents in our study considered it a relevant issue.

### Implementation of recommendations

Despite high awareness of the recommendations, some have been less followed, raising questions about barriers to their implementation. The low rates of preventive information provided may be partly due to time constraints arising from language barriers and service limitations. This may also explain the relatively low rates of mental health assessments, despite studies and reports indicating mental health needs among Ukrainian children (10, 23). Furthermore, the perception that mental health assessments fall within the domain of mental health specialists may have contributed to the low rates of such assessments. Moreover, the European origin of these children and adolescents may have reinforced the perception that their epidemiological risks and previous healthcare experiences were similar to those of the autochthonous population. When asked about reasons for not implementing the recommendations, a small number of respondents stated that the children were well or returning home anyway, making the efforts unnecessary. This may partly explain why, despite awareness of the guidelines, some physicians chose not to address tuberculosis, HIV, Hepatitis C, and other conditions. Assessing the need for tuberculosis, HIV, or hepatitis C screening may also have been challenging due to the requirement for in-depth, potentially time-consuming discussions, further complicated by language barriers and the need to draw blood.

### Strengths and weaknesses

Participation bias may have influenced the results of this study. As the link to the questionnaire was sent only once via an email newsletter covering multiple other topics, participation was, as expected, low. It is quite possible that PCPs most interested in or concerned with the topic were more motivated to participate in the survey. As a result, knowledge of the existing recommendations and adherence to them may be overestimated in this study. Nevertheless, non-responders likely also cared for Ukrainian refugees and faced similar challenges: Refugees in Switzerland are distributed across different regions by the federal government and, within these regions, to different communes as equitably as possible. Primary care pediatricians usually try to accept all children and adolescents in their area or at least within the same zip code. Our findings highlight gaps in the care provided and identify the challenges that PCPs face, indicating areas that need to be addressed to improve care for refugees.

### Implications and recommendations

While the existing clinical recommendations and the likely good awareness of their existence among PCPs represent a first step, the lack of implementation of certain aspects indicates the need for action at multiple levels. PCPs require support and training—for example, training regarding catch-up vaccines and how to deal with missing vaccination charts; migrant health and intercultural care; awareness of differences in epidemiological risks; and the assessment of mental health needs in patients and caregivers with a forced migration background (24).

Gaps identified by our research, such as insufficient screening for communicable diseases, inadequate mental health assessment, and limited preventive care, highlight the need to address the most significant challenges at the health-system level: language barriers and time constraints. While online interpreter tools are sufficient in some cases, the importance of high-quality interpreter services and the gaps related to limited access to such services in Switzerland have been demonstrated previously (25). For adequate interpreter use, rapidly available, high-quality interpreter services that can easily be booked by social services and healthcare providers need to be made available, and healthcare providers must be informed about these new services and trained on how and when to use them (11).

Time constraints were the second most important challenge faced by PCPs, likely affecting the quality of care. Institutional support and political actions regarding an increase in numbers of primary care providers trained are made. In addition, changing the remuneration system for healthcare providers in a way that longer efforts can adequately be billed, and no-shows do not cause financial losses to pediatricians, may also be warranted. On the patient and family side, social institutions can help raise awareness about the importance of attending appointments and provide information on the host country’s health services. Access to mental health and developmental specialists and therapies also needs improvement. There is also a need for more research to foster a better understanding of needs and challenges and to evaluate potential solutions to improve care for refugees. Many of the issues raised are also likely relevant for other refugee groups in Switzerland and Europe (26).

## Conclusion

The reported care provided to Ukrainian minors in Switzerland partially followed the national recommendations. An increase in the provision of preventive information and mental health assessments is particularly desirable from a public health perspective. Gaps in care and the challenges identified need to be addressed by policymakers and healthcare providers. Changes needed in the health system include the following: (1) systematic improvement of interpreter services free of charge, (2) addressing health system constraints affecting care, (3) provision of institutional support, and (4) improved access to mental healthcare. Primary care providers should receive training in migrant health and be involved in the formulation of guidelines and health policies. Refugee care protocols are needed. Qualitative research involving Ukrainian families and primary care physicians would be valuable in improving the understanding of barriers encountered, particularly those related to different expectations, and in further facilitating care.

## Data Availability

The raw data supporting the conclusions of this article will be made available by the authors, without undue reservation.

## Footnotes

1
www.paediatrieschweiz.ch

2
www.findmind.ch
